# Editorial: Advances in the understanding of the affective and cognitive effects of physical activity, exercise, and sports

**DOI:** 10.3389/fpsyg.2024.1383947

**Published:** 2024-03-13

**Authors:** Chong Chen, Yasuhiro Mochizuki, Filipe Manuel Clemente

**Affiliations:** ^1^Division of Neuropsychiatry, Department of Neuroscience, Yamaguchi University Graduate School of Medicine, Ube, Japan; ^2^Center for Data Science, Waseda University, Tokyo, Japan; ^3^Escola Superior Desporto e Lazer, Instituto Politécnico de Viana do Castelo, Rua Escola Industrial e Comercial de Nun'Álvares, Viana do Castelo, Portugal; ^4^Gdansk University of Physical Education and Sport, Gdańsk, Poland; ^5^Sport Physical Activity and Health Research and Innovation Center, Viana do Castelo, Portugal

**Keywords:** aerobic exercise, resistance training, strength training, fitness, executive functions, emotional wellbeing, mental health, polymorphism

Recent research underscores the positive impact of physical activities, exercises, and sports on enhancing cognitive and emotional wellbeing. Improvements have been noted in areas such as attention (De Greeff et al., 2018), executive functions (Hoffmann et al., 2021; Huang et al., 2022), the process of memory and learning (Wanner et al., 2020; Hoffmann et al., 2021), creativity (Rominger et al., 2022; Chen, 2024), the ability to withstand stress (Arida and Teixeira-Machado, 2021; Toth et al., 2023), and mental health (Chen et al., 2017; Chen and Nakagawa, 2023a). Regular participation in physical activities has been linked to a lower incidence of major neurological and psychiatric conditions, including dementia (Chen and Nakagawa, 2023b), major depressive disorders (Harvey et al., 2018), and anxiety disorders (Svensson et al., 2021).

Despite these well-documented benefits, recent research points to unresolved queries (Chen et al., 2022), notably the determination of the optimal type, intensity, and duration of physical activities that maximize benefits across different demographic groups. This gap calls for a deeper dive into individual differences that have been somewhat neglected in past studies. Furthermore, there is an urgent call for the development of detailed strategies to encourage physical activity, a domain still in need of refinement. This Research Topic, through its 18 articles, seeks to address these critical questions, aiming to deepen our knowledge of the cognitive and emotional advantages offered by physical activity.

A focal point of this Research Topic is the investigation into how different aspects of exercise, including its frequency, duration, intensity, and type, influence its effectiveness. One notable contribution is from Lin and Gao, who conducted a meta-analysis of nine randomized controlled trials among college students. Their findings indicate that physical activity interventions significantly reduce anxiety symptoms, demonstrating an effect size of −0.55 in terms of standard mean difference. This analysis highlights that the benefits of exercise on anxiety might be influenced by the exercise's nature, with aerobic exercise and yoga showing effectiveness, whereas resistance training did not yield significant improvements. Furthermore, interventions were notably effective when conducted at least three times weekly, lasting longer than 8 weeks, and at moderate to high intensities. This data aligns with the theory that more vigorous exercise could offer greater mental health and cognitive benefits (Nakagawa et al., 2020), a notion further supported by a study from Gilbert et al. This latter study, although not finding significant cognitive benefits from a single 60-min games-based physical education lesson over a regular academic lesson in adolescents, did observe enhanced working memory in those who engaged in more moderate-to-vigorous physical activity.

These insights, however, bring to light the practical challenges in applying them within clinical settings. Patients with depressive disorders—who often experience concurrent anxiety symptoms (Chen, 2022) and cognitive deficits (Chen et al., 2015)—may have lower physical capabilities and motivation. This dilemma has led to the development of lower-intensity exercise therapies aimed at enhancing participation and satisfaction among patients (Sakai et al., 2021). A potentially optimal approach may involve initiating treatment at lower intensities and incrementally increasing them as patients' physical fitness and motivation improve.

Further expanding on these findings, Liu T. et al.'s cross-sectional survey involving over 67,000 school-age children and adolescents demonstrates that even minimal engagement in sports, as infrequent as 1–3 times a month, can enhance subjective wellbeing. This finding is echoed in the work of Bian and Xiang, who, through a longitudinal study of 10,000 adults, observed that regular participation in sports and exercise clubs, even on a weekly or monthly basis, correlates with a reduced likelihood of depressive symptoms 4 years later. These studies suggest that the threshold of physical activity required for preventing future mental health issues may be lower than what is needed to address existing psychiatric symptoms, as demonstrated in the meta-analysis by Lin and Gao.

Additionally, Liu G. et al. sought to determine the optimal exercise duration for academic performance among ~18,000 Chinese junior high school students. Their research revealed an inverted-U shaped correlation between weekend physical activity and academic achievement, with a noted sex difference in the optimal duration of exercise-−2 h for males and 1 h for females. The inverted-U shape finding aligns with another recent study by Shimura et al. (2023), which examined the association between the total duration of weekly physical activity and mental health outcomes. Moreover, the gender-differentiated impact in the above study is further explored by Wu et al., who found significant associations between physical activity levels and depressive symptoms in males but not in females.

Jin et al.'s study enriches this Research Topic by comparing visual attention among basketball players, swimmers, and non-athletes, revealing that basketball players exhibit superior visual tracking accuracy. This highlights the advantages of open-skill sports, which require constant adaptation to changing environments, in enhancing visual attention and underscores the broader cognitive benefits of specific types of physical activities.

The Research Topic also delves into the specific benefits of physical activity across various populations and disciplines. For instance, Wochyński et al. demonstrated improved physical fitness and cognitive functions in Polish male cadet pilots following 6 months of cadet aviation practice. Moreover, Alhumaid and Said highlighted the positive correlation between physical activity and self-esteem levels in Saudi individuals with physical disabilities.

However, not all studies showcased physical activity's universal benefits. Morava et al. indicated that acute stress's impact on cognitive flexibility might not be alleviated by a single session of vigorous cycling, contrasting with previous evidence that, under stress-free conditions, vigorous cycling may enhance cognitive flexibility (Aga et al., 2021). Moreover, Burns et al. suggested the importance of considering covariates when assessing physical activity's effects on mental health.

The Research Topic also addresses athlete performance enhancement strategies, with Selmi et al. emphasizing mood monitoring in soccer players and Aras et al. investigating the efficacy of mindfulness practices in aiding recovery among female basketball players, albeit without significant findings.

Together, these articles offer a comprehensive perspective on the multifaceted influence of physical activity, suggesting the importance of tailored exercise regimens to optimize affective and cognitive outcomes across diverse populations and settings.

Building on the diverse insights provided by these studies, the Research Topic delves into understanding the factors that influence physical activity behaviors, adding a crucial layer to the narrative. Ma et al. explore how exercise intention and the feelings induced by exercise, such as relaxation, correlate with actual exercise behaviors, noting the moderating role of personality openness in strengthening this relationship. This observation highlights the critical need to consider the emotional responses or mood shifts triggered by exercise when prescribing exercise interventions. Beyond the Exercise-induced Feeling Inventory (Gauvin and Rejeski, 1993) utilized by the researchers, recent concise measures like the Chen-HAgiwara Mood Test (CHAMT, Chen et al., 2024) emerge as promising alternatives. The CHAMT, grounded in the two-dimensional valence-arousal theory of affect and utilizing visual analog scales for a more intuitive assessment than traditional Likert scales, represents a forward-thinking approach to capture the psychological impacts of physical activity (Aga et al., 2021; Matsumoto et al., 2022; Kawashima et al., 2024).

Furthermore, Sheng et al. identify exercise self-efficacy and peer support as key drivers of physical activity levels, while Zhang et al. highlight the pivotal role of the family environment in encouraging physical activity, introducing a novel scale for its assessment. Emphasizing exercise as a family activity, this latter study is in line with the proposal that communal exercise can enhancing bonding, improve adherence to exercise routines and boost overall family wellbeing (Chen, 2017; Koga et al., 2023). This approach promotes incorporating physical activity into family life, underlining its potential to not In addition, Martins et al., in their meta-analysis, highlight the positive correlation between pet ownership and increased physical activity (with an effect size of Cohen's d being 0.55), and Bayraktar et al. suggest genetic factors, such as the rs1800496 polymorphism in the ANKK1 gene, may influence competitive sports involvement.

These studies collectively underscore the complexity of exercise behavior, emphasizing the interplay of psychological, social, environmental, and genetic factors in promoting physical activity. This comprehensive approach not only enriches our understanding of the multifaceted role of physical activity in enhancing mental health, cognitive functions, and academic performance but also highlights the importance of individualized approaches to exercise prescription, catering to the diverse needs and influences affecting different populations.

## Author contributions

CC: Writing – original draft, Writing – review & editing. YM: Writing – review & editing. FC: Writing – review & editing.
